# SVH-BD: Synthetic vegetation hyperspectral benchmark dataset derived from sentinel-2 tiles over France, Spain, Tanzania, and India during 1–6 May 2023

**DOI:** 10.1016/j.dib.2026.112937

**Published:** 2026-06-06

**Authors:** Chedly Ben Azizi, Claire Guilloteau, Gilles Roussel, Matthieu Puigt

**Affiliations:** Univ. Littoral Côte d’Opale, LISIC – UR 4491, EILCO, Campus de la Malassise, CS 50109, F-62698 Longuenesse Cedex, France

**Keywords:** Radiative transfer modelling, Biophysical parameter retrieval, Canopy reflectance simulation, Uncertainty quantification, PROSAIL inversion

## Abstract

This dataset provides a large collection of 10,915 synthetic hyperspectral image cubes paired with pixel‑level vegetation trait maps, designed to support research in radiative transfer emulation, vegetation trait retrieval, and uncertainty quantification. Each hyperspectral cube contains 211 bands spanning 400–2500 nm at 10 nm resolution and a fixed spatial layout of 64 × 64 pixels, offering continuous simulated surface reflectance spectra suitable for emulator development and machine‑learning tasks requiring high spectral detail. Vegetation traits were derived by inverting Sentinel‑2 Level‑2A[1] surface reflectance using a PROSAIL‑based lookup‑table approach[2,3,4], followed by forward PROSAIL simulations to generate hyperspectral reflectance under physically consistent canopy and illumination conditions. The dataset covers four ecologically diverse regions—East Africa, Northern France, Eastern India, and Southern Spain—and includes 5th and 95th percentile uncertainty maps as well as Sentinel‑2 scene classification layers. This resource enables benchmarking of inversion methods, development of fast radiative transfer emulators, and studies of spectral–biophysical relationships under controlled yet realistic environmental variability.

Specifications Table

**Table:** 

Subject	Earth & Environmental Sciences
Specific subject area	Hyperspectral Remote Sensing, Vegetation Trait Retrieval, Radiative Transfer Model Emulation
Type of data	Reflectance hyperspectral images Vegetation Bio-optical parameter maps
Data collection	The dataset was generated through a multi-step pipeline combining satellite data preprocessing, radiative transfer model inversion, and forward hyperspectral simulation. Sentinel-2 Level-2A [1] products were first selected across four geographic regions (East Africa, France, Spain, India). The Sentinel-2 tiles were acquired from Level-2A surface reflectance products via the Harmonised Data Access service, with acquisition dates between 1 and 6 May 2023. The relevant spectral bands were extracted from each Level-2A tile, specifically [‘B1’, ‘B2’, ‘B3’, ‘B4’, ‘B5’, ‘B6’, ‘B7’, ‘B8’, ‘B8A’, ‘B9’, ‘B11’, ‘B12’], and were subsequently cropped and harmonized to obtain spatially consistent multispectral inputs of 64 by 64 pixels, and 20 m ground sampling distance. These inputs were then inverted using a PROSAIL-based look-up table inversion [[2], [3], [4]] to estimate pixel-level vegetation biophysical parameters. Finally, the retrieved parameters were used to drive a forward PROSAIL simulation to produce hyperspectral reflectance cubes in the range of 400–2500 nm.
Data source location	• Institution: Laboratoire d’Informatique, d’Image et du Signal de la Côte d’Opale. • City/Town/Region: Longuenesse. • Country: France
Data accessibility	Repository name: SVH-BD: Synthetic Vegetation Hyperspectral Benchmark Dataset for Emulation of Remote Sensing Images. Data identification number: https://doi.org/10.5281/zenodo.18660571. Direct URL to data: https://zenodo.org/records/18660571
Related research article	None

## 1 Value of the Data

• The dataset provides 10 915 synthetic hyperspectral image cubes with pixel-level vegetation trait maps, offering explicit vegetation trait annotations that are typically unavailable in observational remote sensing archives.
• Each hyperspectral cube includes 211 spectral bands from 400 to 2500 nm at 10 nm resolution, with a fixed spatial resolution of 64×64 pixels and a uniform 20 m ground sampling distance, which ensures spatial consistency across the scenes.
• The forward and inverse modeling steps are guided by plant biophysical constraints, region-specific parameter distributions and soil types. This ensures that simulated reflectance and retrieved traits reflect realistic environmental variability and remain consistent with the ecological characteristics of each region.
• The dataset is specifically designed to support the development of machine learning based hyperspectral emulation for pixel‑wise and spatial-wise settings. Nonetheless, it can also support the evaluation of biophysical parameter retrieval.
• The dataset includes uncertainty maps for vegetation trait retrievals, providing 5th and 95th percentile estimates that support the evaluation of model robustness and the study of uncertainty propagation. Additional Sentinel-2 classification maps are provided for each scene, offering contextual information that supports land-cover-aware analysis, and enables studies that integrate spectral, biophysical, and categorical landscape information.

## 2 Background

The increasing availability of multispectral and hyperspectral satellite missions has made remote sensing indispensable for Earth observation and vegetation monitoring at regional to global scales. Despite this progress, the development of data-driven retrieval and emulation models remains constrained by the limited availability of pixel-level vegetation trait annotations. Large remote sensing archives provide extensive observational coverage but rarely include physically consistent biophysical variables such as chlorophyll content, leaf water content, or leaf area index. This lack of reference data limits the training, benchmarking, and validation of machine learning approaches aimed at linking top-of-canopy reflectance to underlying vegetation properties and observation conditions. To address this gap, physically based simulation has become an essential strategy. In the specific case of vegetation remote sensing, the combined model PROSAIL [4] has been proven popular. PROSAIL combines the PROSPECT leaf optical properties model [2] and SAIL canopy bidirectional reflectance model [2], in order to generate top of canopy reflectance from a combination of plant and soil bio-optical parameters and observation geometry. This allows for a detailed analysis of vegetation characteristics under realistic conditions. Using PROSAIL as a forward model, this dataset has been developed to fill the aforementioned gap, supporting the development of fast emulators that approximate radiative transfer models at significantly reduced computational cost, and providing a benchmark for large scale inversion workflows for vegetation trait retrieval and uncertainty propagation.

## 3 Data Description

The dataset contains 10 915 hyperspectral image cubes, each with spatial dimensions of 64 × 64 pixels and 211 spectral bands. The spectral range spans 400–2500 nm with a fixed 10 nm sampling interval, and all images share a 20 m ground sampling distance (GSD). Each hyperspectral cube is paired with a trait file (traits.tif) containing 16 leaf‑, canopy‑, and observation‑level parameters. Additional files provide uncertainty estimates for trait retrievals (p5.tif, p95.tif) and Sentinel‑2 scene classification maps (quality_scene_classification.img with metadata in quality_scene_classification.hdr). Table 1 provides a summary of the content of each tile folder. Details about traits index mapping can be found in Table 2.

**Table 1:** Summary of files provided for each tile.

Filename	Description
surf-refl.tif	Hyperspectral surface reflectance cube (64×64×211)
traits.tif	Retrieved bio‑optical parameters (16 traits).
p5.tif	5th percentile uncertainty map for trait retrievals.
p95.tif	95th percentile uncertainty map for trait retrievals.
quality_scene_classifcation.img	Sentinel-2 scene classification map.
quality_scene_classifcation.hdr	Metadata associated with the classification map.

**Table 2:** Parameter ranges for the combined leaf (PROSPECT-D) and canopy (4SAIL) RTM (PROSAIL).

Index	Symbol	Description	Unit	Range
Leaf Biochemical Parameters (Prospect-D)				
0	N	Leaf structure parameter	–	[1, 2.5]
1	$C_{ab}$	Leaf chlorophyll a+b content	$\mu \;g/cm^{2}$	[O, 160]
2	$C_{ar}$	Leaf carotenoids content	$\mu \;g/cm^{2}$	[O, 60]
3	$C_{ant}$	Leaf anthocyanins content	$\mu g/cm^{2}$	[0, 5]
4	$C_{brown}$	Brown pigments	–	[0, 0.1]
5	$C_{w}$	Equivalent water thickness	$g/cm^{2}$	[0, 0.07]
6	$C_{m}$	Dry matter content	$g/cm^{2}$	[0, 0.1]
Canopy Structural Parameters (SAIL)				
7	LAI	Leaf Area Index	–	[0, 10]
8	$LIDF_{a}\left(ALA\right)$	Average leaf angle	degrees	[30, 70]
9	$LIDF_{b}$	Bimodality	–	0
14	TypeLIDF	leaf inclination distribution function	-	1
10	$h_{spot}$	Hot spot parameter	–	[0.01, 0.5]
Observation Geometry				
11	$\theta_{s}$	Solar zenith	degrees	[15, 80]
12	$\theta_{v}$	Vien zenith	degrees	[0, 35]
13	ϕ	Relative azimuth	degrees	[100, 150]
Soil Parameters				
15	$\rho_{soil}$	Soil spectrum	–	Library (ICRAF)

**Fig. 1:**
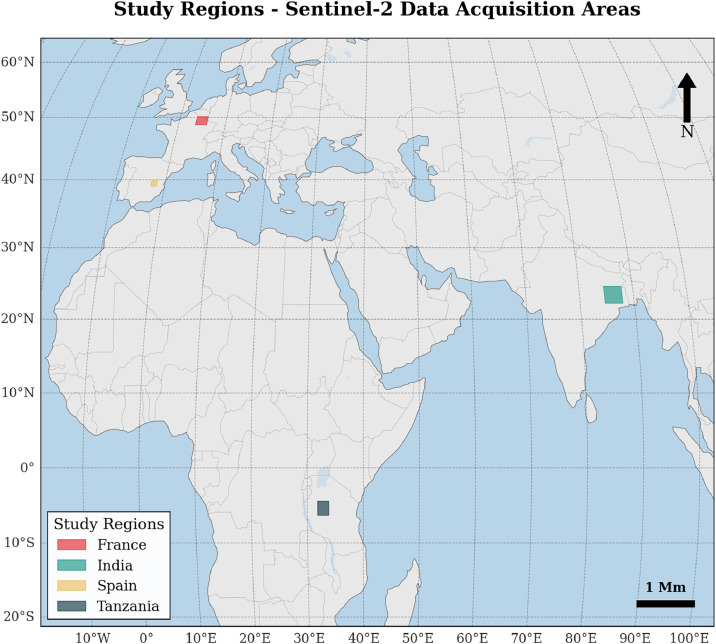
Geographic distribution of the four study regions used for synthetic hyperspectral dataset generation.

The dataset is organized by geographical region (Africa, France, India, Spain). Within each region, subfolders correspond to tile identifiers as shown in Fig. 1.

## 4 Experimental Design, Materials and Methods

The dataset was generated through a multi-step pipeline combining satellite data preprocessing, radiative transfer model inversion, and forward hyperspectral simulation. Sentinel-2 Level-2A [1] products were first selected and harmonized to obtain spatially consistent multispectral inputs across four geographic regions. These inputs were then inverted using a PROSAIL-based look-up table to estimate pixel-level vegetation biophysical parameters. Finally, the retrieved parameters were used to drive a forward PROSAIL simulation to produce hyperspectral reflectance cubes.

### 4.1 Sentinel 2 data preprocessing and image selection

Sentinel-2 is a multispectral Earth observation mission operated by the European Space Agency (ESA) within the Copernicus Programme. It consists of twin satellites (S2A and S2B) equipped with the MultiSpectral Instrument (MSI), which provides imagery across 13 spectral bands spanning the. visible to shortwave infrared regions. These data are widely used for vegetation monitoring, land cover mapping, and retrieval of biophysical and biochemical parameters. The MSI acquires data at three spatial resolutions—10 m, 20 m, and 60 m—depending on the spectral band configuration. The Sentinel-2 tiles used for this dataset were acquired from Level-2A surface reflectance products (S2MSI2A) via the Harmonised Data Access service. Four regions—East Africa (Tanzania), Northern France, Spain, and Eastern India—were selected to provide diverse vegetation types and environmental conditions. Scene selection was performed using spatio-temporal queries specifying geographic bounding boxes (Fig. 1) and acquisition dates between 1 and 6 May 2023. All scenes corresponded to platform S2A, instrument MSI, and ONLINE data status. After retrieval, a harmonizing preprocessing pipeline was applied to ensure spectral and spatial consistency across all products. The relevant spectral bands were extracted from each Level-2A tile, specifically [‘B1’, ‘B2’, ‘B3’, ‘B4’, ‘B5’, ‘B6’, ‘B7’, ‘B8’, ‘B8A’, ‘B9’, ‘B11’, ‘B12’]. The 20 m tiles - bands 1–8, bands 11 and 12 - were cropped into patches of 64×64pixels, while the 60 m tiles - band 9- were initially cropped at 32×32 pixels and subsequently upsampled to 64×64 using nearest neighbour interpolation to maintain uniform spatial resolution across all bands. The resulting 10,883 multispectral cubes are inverted in the next step in order to obtain leaf and canopy biophysical parameters using PROSAIL.

### 4.2 Radiative transfer modelling and look-up table inversion

The dataset was generated using a two-stage radiative transfer modeling (RTM) pipeline designed to simulate hyperspectral canopy reflectance spectra from vegetation biophysical parameters. This framework integrates two physically based models —PROSPECT-D [2,4] and SAIL [3]— which model light interactions at the leaf and canopy scales, respectively.

### 4.3 Leaf-Level simulation: prospect-d

The PROSPECT-D model simulates leaf reflectance and transmittance as a function of biochemical and structural leaf properties. The model requires six input parameters: the leaf structure parameter N (number of effective layers), chlorophyll a+b content $C_{ab}\left(\mu \;g/cm^{2}\right)$, carotenoid content $C_{ar}\left(\mu \;g/cm^{2}\right)$, anthocyanin content $C_{ant}\left(\mu g/cm^{2}\right)$, brown pigment content $C_{brown}$ (dimensionless), equivalent water thickness $C_{w}\left(g/cm^{2}\right)$, and dry matter content $C_{m}\left(g/cm^{2}\right)$. These parameters collectively describe the optical behavior of leaves across the visible to shortwave infrared spectrum.

### 4.4 Canopy-level simulation: SAIL

At the canopy level, the SAIL model integrates the outputs of PROSPECT-D with structural and environmental variables to simulate canopy reflectance. The required canopy parameters include the leaf area index LAI (dimensionless), average leaf inclination angle (ALA, degrees), a hotspot parameter controlling directional reflectance, and the fraction of diffuse incoming radiation. Observation geometry (solar zenith, viewing zenith, and relative azimuth angles) is included to capture bidirectional reflectance effects. Finally, soil spectra are supplied for each selected region under the assumption of within-region homogeneity, drawn from the ICRAF-ISRIC soil spectral database [5] using the nearest available measurement site to the target area. The model also computes auxiliary variables such as the fraction of absorbed photosynthetically active radiation $f_{APAR}$ and albedo, which are key indicators of canopy-level energy exchange.

### 4.5 RTM inversion

To generate the synthetic hyperspectral dataset, Sentinel-2 surface reflectance images were inverted using a lookup-table (LUT) derived from the PROSAIL model to estimate spatially coherent maps of vegetation biophysical parameters. The LUT contained M=50000 simulated spectra generated by sampling the parameter space defined in Table 2. Parameter values were drawn using Latin Hypercube Sampling (LHS) to ensure a homogeneous and statistically efficient exploration of the multidimensional space. For a parameter $\theta_{j}\;$defined over $\left[\theta_{j}^{min},\theta_{j}^{max}\right]$, the i-th LHS sample was computed as.

$${\theta_{j}}^{\left(i\right)}={\theta_{j}}^{min}+\frac{\pi_{j}\left(i\right)-{U_{j}}^{\left(i\right)}}{M}\left({\theta_{j}}^{max}-{\theta_{j}}^{min}\right),$$

where $\pi_{j}$ is a random permutation of {1,2,…,M} and ${U_{j}}^{\left(i\right)}∼U\left(0,1\right)$ is a uniform random variable.

Physiological constraints were applied during LUT construction following [6], notably the empirical coupling between chlorophyll content ($C_{ab}$) and leaf area index (LAI), ensuring that sampled parameters corresponded to realistic vegetation states. Additional plausibility checks were performed at the spectral level: simulated spectra exhibiting green peaks at wavelengths lower than 547 nm were removed, following the criterion of [7]. Each retained LUT entry consisted of a parameter vector $\theta^{\left(i\right)}$ and its corresponding PROSAIL-simulated reflectance spectrum $\rho^{\left(i\right)}\left(\lambda \right)$. The inversion step consisted of matching observed Sentinel-2 reflectance $\rho^{obs}$ to the simulated LUT entries. For each pixel, spectral discrepancy was quantified using the root mean square error (RMSE) as a cost function,

$$RMSE\;=\;\sqrt{\frac{1}{211}\sum_{b=1}^{211}{\left({\rho_{b}}^{obs}-{\rho_{b}}^{sim}\right)}^{2}}.$$

For each pixel, n=10 LUT entries with the lowest cost values were retained, forming an ensemble of plausible biophysical solutions. Final parameter estimates were derived using the median. Parameter uncertainty was assessed non-parametrically from the 5th and 95th percentiles of the n-best ensemble, providing pixel-level confidence intervals. The resulting spatially explicit maps of PROSAIL biophysical parameters were then used as inputs to a forward PROSAIL simulation chain to generate pixel-level hyperspectral reflectance spectra. Each simulated spectrum represents canopy-scale reflectance under the geometric, physiological, and structural conditions estimated during the inversion.

### 4.6 Visualisation

**Fig. 2:**
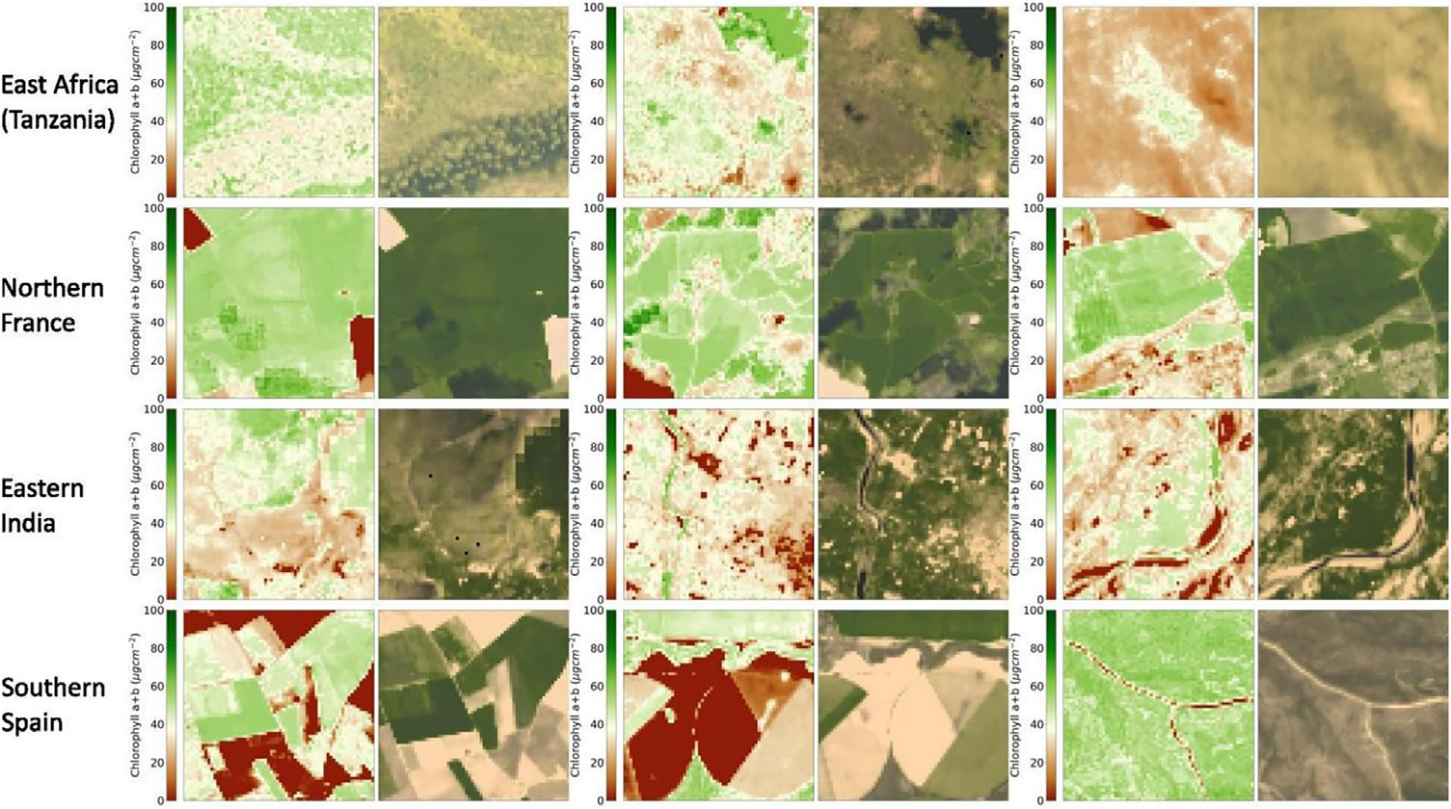
Representative examples of simulated hyperspectral image cubes and their corresponding chlorophyll a+b ($C_{ab}$) maps from the four study regions. For each row, the left column shows the $C_{ab}$ map and the right column shows the associated HSI scene. All scenes are displayed using an RGB composite. Ground sample distance (GSD) is 20 m.

**Fig. 3:**
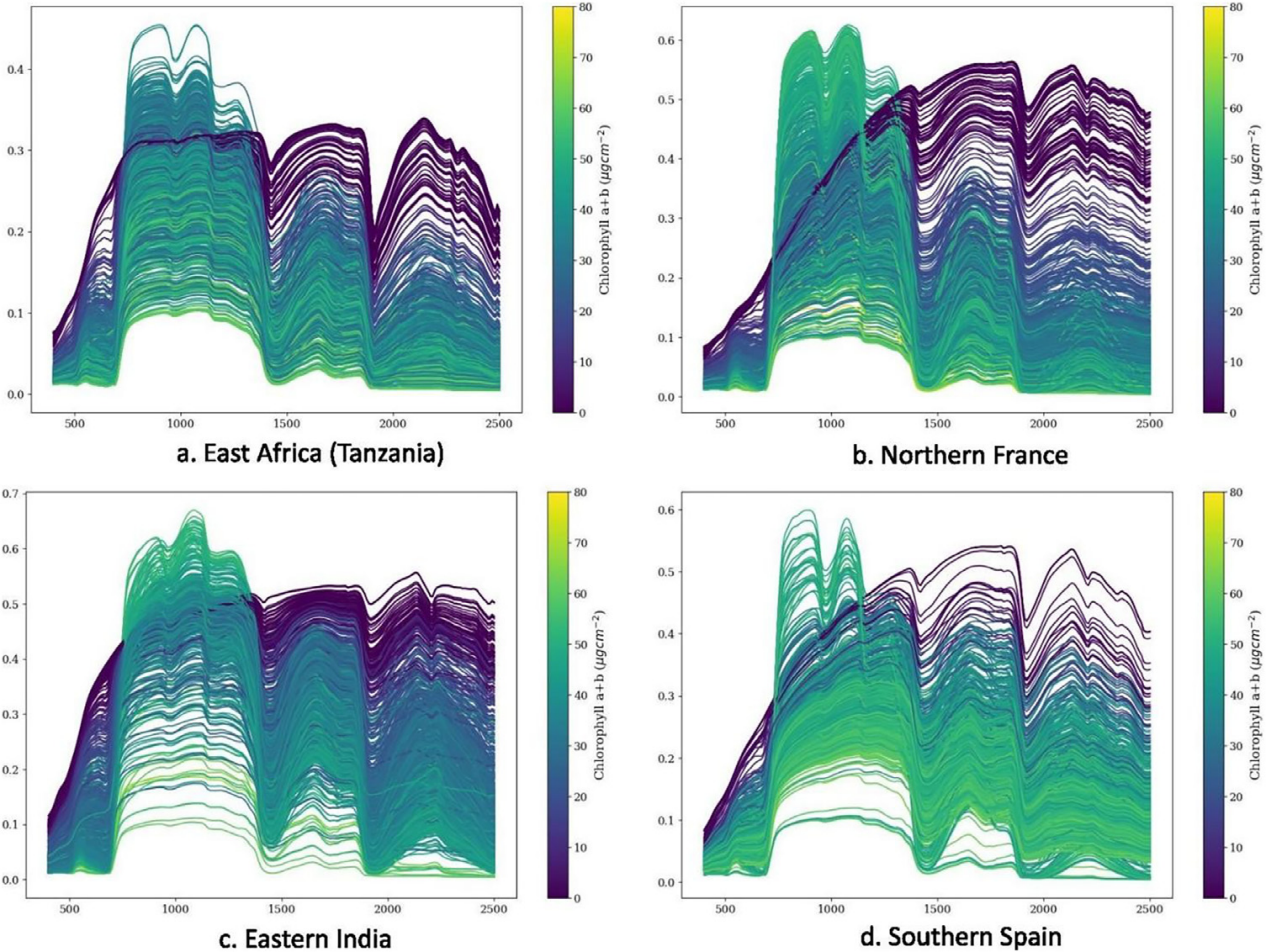
Simulated hyperspectral reflectance spectra from the four study regions, color-coded by chlorophyll a+b content ($C_{ab}$).

Representative examples of HSI cubes from each region are shown in Fig. 2. These samples illustrate the visual diversity of the dataset in terms of canopy cover, landscape structure, and spectral signatures. For each region, the figure displays the chlorophyll a+b ($C_{ab}$) map alongside its corresponding RGB composite HSI scene, allowing simultaneous inspection of biochemical variability and spatial reflectance patterns. The examples highlight transitions between cropland parcels, forested clusters, semi-arid shrublands, and heterogeneous mosaics characteristic of the selected environments. In addition, the spectral behavior associated with these land-cover configurations is illustrated in Fig. 3, which shows representative reflectance spectra for each region, color-coded by $C_{ab}$ concentration to emphasize the relationship between vegetation biochemistry and spectral shape.

## Limitations

While the dataset covers four ecological regions, it does not capture the full diversity of global vegetation or environmental conditions. One notable omission are the Americas, which are not represented in this work. Additionally, due to the limited time coverage of the original Sentinel 2 tiles, seasonal change is not taken into account, which may limit the ability of models trained on this dataset to generalize across seasons. Furthermore, each region is represented using a single soil type, which does not reflect intra-regional soil variability and therefore the full range of soil-driven effects on bio-optical parameters. Moreover, the synthetic generation process also relies on strong assumptions in both the forward and inverse modeling steps —namely a fixed leaf inclination distribution function and pure or weakly mixed pixels— which should be considered when applying the dataset beyond controlled experimental settings. Finally, the resolution of the simulated dataset is limited by the resolution of the Sentinel-2 inputs. This may limit the use of the dataset in applications requiring fine spatial details or involving sub-pixel heterogeneity. Pixels that don’t represent soil or vegetation should be masked using the provided Sentinel-2 classification maps.

## Ethics Statement

The authors have read and follow the ethical requirements for publication in Data in Brief. The current work does not involve human subjects, animal experiments, or any data collected from social media platforms.

## CRediT Author Statement

**Chedly Ben Azizi:** Conceptualization, Methodology, Software, Validation, Data Curation, Writing – Original Draft. **Claire Guilloteau:** Conceptualization, Validation, Writing – Reviews & Editing, Supervision, Funding acquisition. **Gilles Roussel:** Conceptualization, Validation, Writing – Reviews & Editing, Supervision, Funding acquisition. **Matthieu Puigt:** Writing – Reviews & Editing.

## Data Availability

ZenonoSVH-BD : Synthetic Vegetation Hyperspectral Benchmark Dataset for Emulation of Remote Sensing Images (Original data)

## References

[1] Modified Copernicus sentinel data 2023/Sentinel Hub, https://www.sentinel-hub.com/.

[2] Jacquemoud S., Baret F. (1990). PROSPECT: a model of leaf optical properties spectra. Remote Sens. Environ..

[3] Verhoef W. (1984). Light scattering by leaf layers with application to canopy reflectance modeling: the SAIL model. Remote Sens. Environ..

[4] Jacquemoud S. (2009). PROSPECT+ SAIL models: a review of use for vegetation characterization. Remote Sens. Environ..

[5] World Agroforestry (ICRAF) and International Soil Reference and Information Centre (ISRIC) (2021).

[6] Danner M. (2021). Efficient RTM-based training of machine learning regression algorithms to quantify biophysical & biochemical traits of agricultural crops. ISPRS J. Photogram. Remote Sens..

[7] Wocher M. (2020). RTM-based dynamic absorption integrals for the retrieval of biochemical vegetation traits. Int. J. Appl. Earth Observ. Geoinform..

